# Cardiac leiomodin2 binds to the sides of actin filaments and regulates the ATPase activity of myosin

**DOI:** 10.1371/journal.pone.0186288

**Published:** 2017-10-12

**Authors:** Dávid Szatmári, Beáta Bugyi, Zoltán Ujfalusi, László Grama, Réka Dudás, Miklós Nyitrai

**Affiliations:** 1 University of Pécs, Medical School, Department of Biophysics, Pécs, Hungary; 2 University of Pécs, Szentágothai Research Centre, Pécs, Hungary; 3 Hungarian Academy of Sciences-University of Pécs, Nuclear-Mitochondrial Interactions Research Group, Pécs, Hungary; University of Heidelberg Medical School, GERMANY

## Abstract

Leiomodin proteins are vertebrate homologues of tropomodulin, having a role in the assembly and maintenance of muscle thin filaments. Leiomodin2 contains an N-terminal tropomodulin homolog fragment including tropomyosin-, and actin-binding sites, and a C-terminal Wiskott-Aldrich syndrome homology 2 actin-binding domain. The cardiac leiomodin2 isoform associates to the pointed end of actin filaments, where it supports the lengthening of thin filaments and competes with tropomodulin. It was recently found that cardiac leiomodin2 can localise also along the length of sarcomeric actin filaments. While the activities of leiomodin2 related to pointed end binding are relatively well described, the potential side binding activity and its functional consequences are less well understood. To better understand the biological functions of leiomodin2, in the present work we analysed the structural features and the activities of *Rattus norvegicus* cardiac leiomodin2 in actin dynamics by spectroscopic and high-speed sedimentation approaches. By monitoring the fluorescence parameters of leiomodin2 tryptophan residues we found that it possesses flexible, intrinsically disordered regions. Leiomodin2 accelerates the polymerisation of actin in an ionic strength dependent manner, which relies on its N-terminal regions. Importantly, we demonstrate that leiomodin2 binds to the sides of actin filaments and induces structural alterations in actin filaments. Upon its interaction with the filaments leiomodin2 decreases the actin-activated Mg^2+^-ATPase activity of skeletal muscle myosin. These observations suggest that through its binding to side of actin filaments and its effect on myosin activity leiomodin2 has more functions in muscle cells than it was indicated in previous studies.

## Introduction

The power of muscle contraction depends on the proper interactions of sarcomeric actin-based thin and myosin-based thick filaments [1, 2]. Actin filament remodeling is important for striated muscle function. The organisation and dynamics of sarcomeric actin filaments—which is essential for force generation—are regulated by a large repertoire of actin binding proteins.

Leiomodins (Lmods) are vertebrate members of the tropomodulin (Tmod) gene family, which were recently recognised as regulators of muscle functioning. Lmods are expressed in different muscle tissues [3]. Lmod1 can be found in smooth muscle [4, 5] and was recently reported as a megacyst microcolon intestinal hypoperistaltis syndrome (MMIHS) disease gene in humans and mice [6]. Transcripts encoding the *LMOD2* gene are present in fetal and adult heart and also in adult skeletal muscle [7–9]. The human *LMOD2* gene is located near the hypertrophic cardiomyopathy locus *CMH6* on human chromosome 7q3, potentially implicating this protein in the disease [3]. The third form, the fetal Lmod3 is required for embryonic myofibrillogenesis and implicated in nemaline myopathy in both humans and mice [10–12].

**Fig 1:**
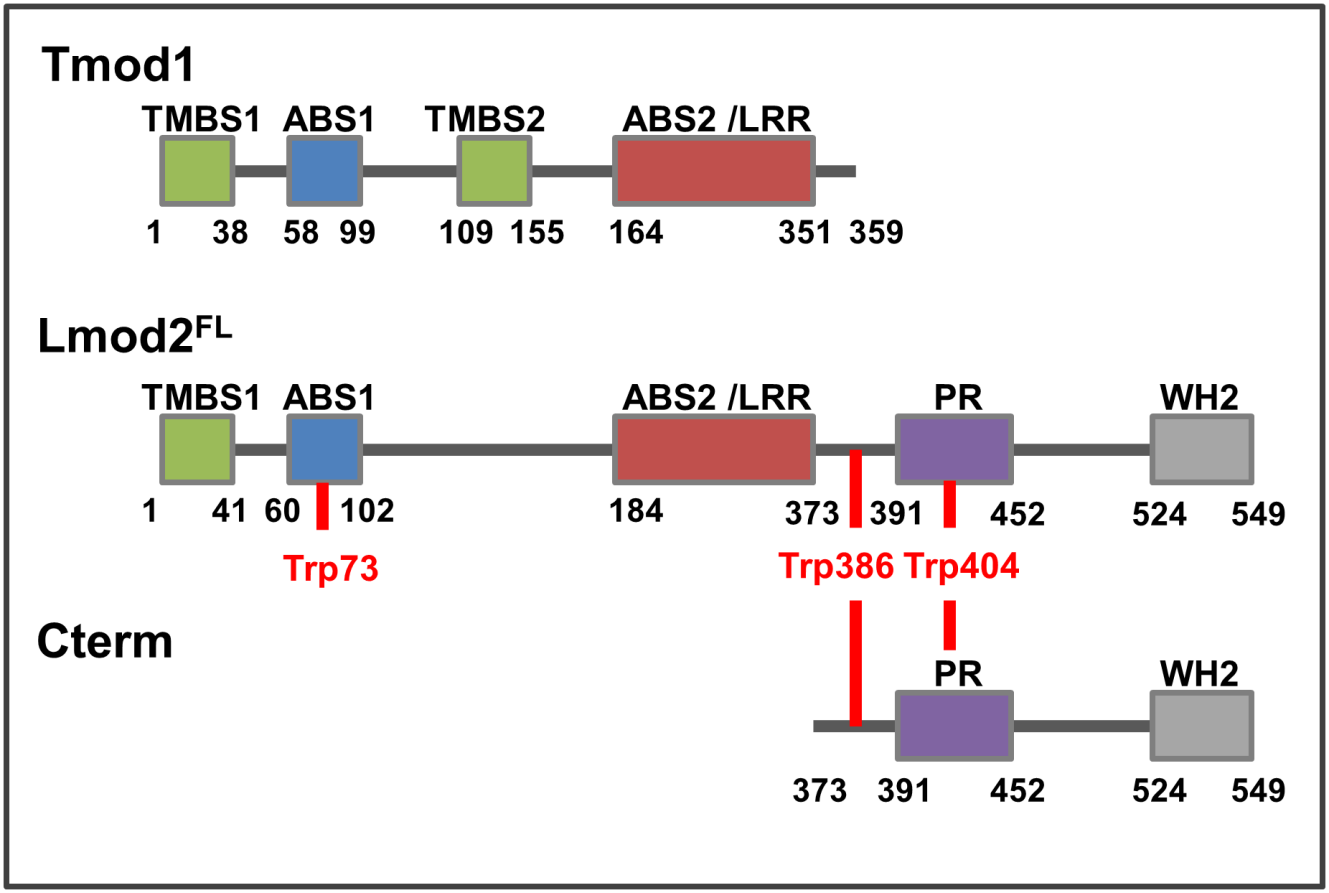
Domain structure of human tropomodulin1 and *Rattus norvegicus* cardiac leiomodin2. The scheme compares the domain organisation of Tmod1 to the full length (Lmod2^FL^) and the C-terminal region (Cterm) of cardiac leiomodin2. Tmod1 and Lmod2^FL^ contain different tropomyosin binding domains (TMBS1/2), homolog actin binding domains (ABS1) and leucine-rich repeats (ABS2/LRR). Lmod2 contains an additional C-terminal extension (Cterm) comprising a proline-rich (PR) and a Wiskott–Aldrich syndrome protein (WASP)–homology 2 (WH2) domain. Tryptophans used in spectroscopy experiments are marked with red.

Tropomodulins and leiomodins built up by homolog domain structures (Fig 1). Both proteins contain different tropomyosin binding domains (TMBS1/2), an actin binding domain (ABS1) and a leucine-rich repeat (ABS2/LRR) that binds monomeric actin as well [7, 13, 14]. In addition to these homolog domains, the structure of Lmods diverge from that of Tmods by possessing a C-terminal extension (Cterm), which contains a proline-rich region (PR, a potential recognition site of intracellular signalling), helical domains and a Wiskott–Aldrich syndrome protein (WASP)–homology 2 (WH2) domain [7, 13, 15].

In cardiac sarcomeres the expression of Lmod2 and Tmod1 depends on the maturation stage of myofibrils. Tmod1 is associated to actin filaments at the early stages of myofibril assembly, before the striated pattern is established [16]. Lmod2 appears later and its expression correlates with the development of myofibrils *in situ* and in some cases it associates with sarcomeres in matured cardiac muscle [8]. Depletion or overexpression of either of these proteins compromises sarcomeric thin filament length and organisation. Overexpression of Tmod1 caused the early lethality of embryos or deletion of Lmod2 caused death of juveniles by dilated cardiomyopathy [8, 9, 17], suggesting that they regulate sarcomeric actin dynamics through different mechanisms. Both proteins localize to the pointed end of actin filaments [8, 18, 19]. However it was proposed that their localisation is manifested through fundamentally different mechanisms to the pointed ends of two distinct subsets of actin filaments in cardiac myofibrils [14]. While Tmods inhibit sarcomeric actin filament lengthening by capping pointed ends [18, 20, 21], Lmod2 maintains pointed end dynamics and antagonises to the capping effect of Tmod [8, 14]. Based on their effects on thin filament length *in vivo*, Lmods were proposed to act as actin filament nucleators in muscle cells [7, 14]. Indeed, they accelerate actin assembly *in vitro* [7, 11, 14], however, the nucleating ability of Lmods has not been demonstrated *in vivo*. Chicken Lmod2 expression is first detected in the heart after it has started to beat [8], indicating that this protein plays a role in the regulation of thin filament lengthening during the maturation of the heart tissues, rather than in initial filament nucleation. According to the recently proposed model of human cardiac Lmod2-actin interaction [13], Lmod2 can bind three actin monomers simultaneously through its ABS1/2 and WH2 motifs (Fig 1). Efficient enhancement of actin polymerisation requires both the N-terminal ABS and the C-terminal WH2 domains [7, 14]. The WH2 domain alone is not sufficient for actin nucleation, in agreement with recent views [22, 23]. The WH2 domain of Lmod2 seems to be required for filament elongation because its removal results in shorter actin filaments in rat cardiomyocytes, suggesting pointed end capping by isolated N-terminal of Lmod2, similarly to Tmod1 [8]. Lmods interact with tropomyosin (Tpm) in an isoform specific manner and this interaction affects the actin polymerisation promoting effect of Lmods [7, 24–26]. It was suggested that tropomyosin modifies the pointed-end interaction but not the *de novo* nucleation activity of cardiac Lmod2, which can be explained by different structural compatibilities [8, 13]. Considering the functional and structural differences between Tmod1 and Lmod2, one can hypothesise that the members of tropomodulin gene family were possibly developing with exon duplications and deletions, thus their evolution shows similarities with fuzzy proteins [27]. Both *in vitro* and *in vivo* observations support that one of the main functions of Lmod and Tmod proteins is related to pointed end binding, through this interaction they tune the length of the thin filaments in a competitive fashion to maintain the final organisation and sarcomere architecture.

Interestingly, while Tmod1 localises only to pointed ends towards the M-lines, Lmod2 can be found near the M-lines and also shows diffuse distribution along the entire length of the thin filaments in rat cardiomyocytes, which relies on its ABP2/LRR domain [8, 14]. A previous study reported that the expression and sarcomeric localisation of Lmod are enhanced during myofibril maturation [28]. Immunofluorescent images of three days old rat cardiac cells show comparable fluorescent emission intensities of actin as leiomodin. Not only the enhanced level of Lmod-GFP localises along the thin filaments but anti-Lmod antibody as well relates to an elevated physiological expression level of leiomodin along the thin filaments distinct from M-lines in matured chicken cardiac cells and zebrafish skeletal muscle cells. Cellular localisation of Lmod can be labelled at both sides from myomesin in M-lines, and it is well separated from α-actinin in Z-disks, frequently flanks single or double bands of Tmod, and partially overlaps with the myosin thick filaments in matured cardiac sarcomeres. Probably, any extra- or intracellular influence can modify the localisation of leiomodin and its expression depends on the maturation level and actual state of muscle cells. This suggests that Lmods bind directly to the sides of thin filaments and besides the pointed end-binding related functions Lmods may regulate other aspects of sarcomeric thin filament function/organisation through their side-binding ability.

In this work our interest is focused on the better understanding of the function and molecular role of cardiac leiomodin2 in sarcomeric actin filament regulation. We expressed the full length *Rattus norvegicus* cardiac Lmod2 (Lmod2^FL^) and a C-terminal fragment possessing only the WH2 actin binding module (Lmod_373-549_, Cterm) (Fig 1). We characterised their intrinsic structural features by steady-state and time-dependent fluorescence spectroscopy. We investigated the actin assembly efficiencies of different regions of Lmod2 in polymerisation assays. We also addressed the side binding activity of leiomodin2 and its potential functional consequences using high-speed sedimentation and fluorescence spectroscopic approaches.

## Materials and methods

### Leiomodin expression and purification

*Rattus norvegicus* full length cardiac Lmod2 (NP_001094434.1; Lmod2^FL^) and the C-terminal fragment (373–549 aa; Cterm) were expressed and purified using the Twin-CN (NE BioLabs) chitin-intein self-cleavage and purification system. DNA constructs were obtained from Roberto Dominguez’s lab and cloned into pTyB1 vectors. Fusion proteins containing chitin-binding and intein tags were expressed for 20 hours at 20°C in ER2566 *E*.*coli* cells. Cells were harvested by centrifugation (6000 g, 10 min, 4°C) then lysed with sonication in Column Buffer (20 mM TRIS, 500 mM NaCl, pH 8.5). The supernatant of the ultracentrifuged (100.000 g, 1 h, 4°C) sample was loaded onto a chitin column, where intein self-cleavage was induced by a thiol reaction using 50 mM DTT, followed by the elution of the target protein from the column. Lmod2^FL^ and Cterm concentrations were determined by either measuring the absorption spectra (Jasco V-550 spectrophotometer) and using the extinction coefficients of 23950 M^-1^cm^-1^ and 12490 M^-1^cm^-1^ at 280 nm, respectively or with the Bradford Protein assay (Bio-Rad). The two methods gave identical results.

### Protein preparation and labelling

Rabbit skeletal muscle actin was prepared from acetone powder as described earlier by Spudich and Watt [29], and stored in bufferA (2 mM TRIS, 0.2 mM ATP, 0.1 mM CaCl_2_, 0.1 mM β-mercaptoethanol, pH 8.0). Fluorescent labelling was carried out in β-mercaptoethanol free bufferA. Cys374 of actin was labelled with either IAEDANS (5-((((2Iodoacetyl)amino)ethyl)amino)Naphthalene-1-Sulfonic Acid) as a FRET donor or IAF (5-Iodoacetoamidofluorescein) as FRET acceptor as described earlier [30–32]. Pyrene (N-1-pyrene-iodoacetamide) labelling was carried out by a standard protocol as described earlier [33]. Actin concentration and labelling ratios were determined from the absorption spectra (Jasco V-550 spectrophotometer). The labelling ratios were 0.8–0.9 for IAEDANS, 0.7–0.8 for IAF and 0.6–0.8 for pyrene. Skeletal muscle tropomyosin (Tpm1.1/2.2) [34] was purified as described [35, 36].

### Characterisation of the fluorescence of intrinsic tryptophan

The Lmod2^FL^ contains 3 tryptophans (Fig 1): one in the N-terminal ABS1 (W73) and two in the C-terminal extension (W386, W404). These tryptophans were used as intrinsic probes for the fluorescence emission coupled structural dynamics measurements of Lmod2. The structural change of chains neighbouring tryptophan can affect their excitation and emission spectra. Fluorescence spectra were measured with Perkin Elmer LS 50B fluorimeter (λ_ex_ = 282 nm, λ_em_ = 354 nm).

### Steady-state and time-dependent anisotropy measurements

The steady-state anisotropy of intrinsic tryptophans in Lmod2^FL^ (9 μM) and Cterm (4.8 μM) fragments was measured with Horiba Jobin Yvon spectrofluorimeter (λ_ex_ = 282 nm, λ_em_ = 354 nm). Fluorescence lifetime and anisotropy decay of the intrinsic tryptophan of Lmod2^FL^ and Cterm were measured using the cross-correlation phase-modulation method (ISS K2 multi-frequency phase fluorimeter) [31]. The excitation and emission wavelengths were 280 nm and 350 nm, respectively. The emission polarizer was set to 0° and 90° as compared to the excitation polarizer to measure I_VV_ (t) and I_VH_ (t), where V and H subscripts refer to vertical and horizontal setting of excitation and emission polarizers, respectively. The instrument response function (IRF) was determined by measuring light scattering of a glycogen solution. The G value was determined by measuring the ratio of I_HV_ (t) / I_HH_ (t). The intensity decay data were analysed assuming the following multi-exponential decay law:

$$I_{total}\left(t\right)=I_{VV}\left(t\right)+2I_{VH}\left(t\right)=\sum_{i}a_{i}\text{exp}\left(-t/\tau_{i}\right)$$ (1)

where a_i_ and τ_i_ are the normalised pre-exponential factors and decay times, respectively. The average fluorescence lifetimes were found to be τ_Lmod2FL_ = 2.92 ± 0.24 ns and τ_Cterm_ = 3.26 ± 1.41 ns for Lmod2^FL^ and Cterm, respectively. Anisotropy values were calculated using Eq 2:

$$r\left(t\right)=\frac{I_{VV}\left(t\right)-I_{VH}\left(t\right)}{I_{VV}\left(t\right)+2I_{VH}\left(t\right)}$$ (2)

Anisotropy decay data were analysed using Eq 3:

$$r\left(t\right)=\sum_{i}r_{0i}\text{exp}\left(-t/t_{i}\right)$$ (3)

where r_oi_ are fractional anisotropies, which decay with rotational correlation time of t_i_. Steady-state anisotropy values correspond to the rotational diffusion of the protein containing fluorophores, which reflect the rotational movement of the whole protein and the flexibility of protein chains containing tryptophan. Rotational correlation times derived from anisotropy decay measurements depend on the mobility of tryptophan, as well as on the protein chains.

### Actin polymerisation assays

To study actin polymerisation, the time dependent fluorescence intensity was measured using pyrene labelled actin. The actin concentration was 4 μM containing 5% pyrene actin. The effect of Lmod2 constructs were investigated at different salt conditions; low salt (0.5 mM MgCl_2_, 10 mM KCl); medium salt (1 mM MgCl_2_, 50 mM KCl); high salt (2 mM MgCl_2_, 100 mM KCl). The final ionic strength (IS) at each condition was derived using the following equation:

$$\left[\text{IS}\right]=\frac{1}{2}\sum_{\text{i}=1}^{\text{n}}{\text{c}}_{\text{i}}{\text{z}}_{\text{i}}$$ (4)

where c_i_ and z_i_ are the molar concentrations and charge of the ions, respectively. The polymerisation rates were determined in the absence or presence of Lmod2^FL^ and Cterm from the slope of the pyrene curves at 50% of maximal change. Normalised polymerisation rate was calculated as the ratio of the slope obtained in the presence of Lmod2 constructs to the slope determined for spontaneous actin assembly. Statistical analysis was performed using Student’s t-test (Microsoft, Excel).

#### Critical concentration measurements

To determine the critical concentration of actin assembly, we measured the fluorescence emission of 0.03; 0.05; 0.1; 0.3; 0.5; 0.7; 1; 3; and 5 μM actin (5% pyrene labelled) in the absence or presence of Lmod2^FL^ (100 nM) or Cterm (4 μM) under high salt conditions. The critical concentration, corresponding to the free G-actin concentration at steady-state was determined from the actin concentration ([a]) dependence of the pyrene fluorescence emission (F) using Eq 5.

$$\text{F}={\text{F}}_{\text{c}}+\left[\left({\text{L}}_{\text{s}}+{\text{R}}_{\text{s}}\right)\frac{\left[\text{a}\right]-\text{cc}}{2}\right]-\left[\left({\text{L}}_{\text{s}}-{\text{R}}_{\text{s}}\right)\frac{\left[\text{a}\right]-\text{cc}}{2}\right]$$ (5)

where F_c_ is the fluorescence emission corresponding to the critical concentration, L_s_ and R_s_ are the slopes of the function below and over the critical concentration, respectively, and c_c_ is the critical concentration.

### High-speed cosedimentation assays

F-actin (4 μM) was incubated with Lmod2^FL^ under medium and high salt conditions. Samples were centrifuged at 258,000 g for 30 min at room temperature. The pellets were resuspended and analysed by SDS-PAGE. The band volumes were derived by GeneTools software (Synaptics Ltd.) and corrected for the molecular weight of the proteins and for the staining efficiency of Coomassie blue. The ratio of the MW corrected band intensities of pelleted Lmod2^FL^ to pelleted actin was derived (protein ratio, y) and plotted as a function of total Lmod2^FL^ concentration. Data was fitted with sigmoidal function:

$$y=\frac{y_{min}-y_{max}}{1+\text{exp}\left(x-x_{0}\right)/dx}+y_{max}$$ (6)

where y_min_ and y_max_ are the protein ratios in the absence and presence of saturating amount of Lmod2, respectively, x is the total Lmod2 concentration, x_0_ is the Lmod2 concentration corresponding to the inflection point and dx is the slope of the linear segment of the sigmoidal.

### Fast kinetic measurements of the interactions of Lmod2 with actin filaments

To measure the binding kinetics of leiomodin to F-actin the real time change of pyrene F-actin fluorescence (5% pyrene labelled) mixed with leiomodin under medium salt conditions was measured by a stopped-flow fast kinetic system (Applied Photophysics Ltd.). The change in pyrene fluorescence as a function of time (y(t)) was fitted using the following function:

$$y\left(t\right)=A_{1}\left(1-exp\left(-\frac{t}{t_{1}}\right)\right)+A_{2}\left(1-exp\left(-\frac{t}{t_{2}}\right)\right)$$ (7)

where A_1_ and A_2_ are the amplitude of the first and second exponential, respectively, t_1_ and t_2_ are the corresponding times.

### Inter-monomer FRET measurements

Actin monomers labelled separately with IAEDENS (donor) or with IAF (acceptor) were mixed in a donor/acceptor ratio of 1: 10 for FRET measurements [30, 37] then polymerised to filaments under medium salt conditions. To investigate the Lmod induced structural changes in F-actin, the temperature-dependent inter-monomer FRET efficiency of IAEDENS-IAF F-actin (4 μM) was measured in the presence or absence of Lmod2^FL^ (5 μM) (Horiba Jobin Yvon spectrofluorimeter, λ_ex_ = 350 nm, λ_em_ = 380–600 nm). The temperature was set between 5–35°C. Spectra were corrected for the inner filter effect and the emission of the donor in the absence (F_D_) or presence (F_DA_) of acceptor was derived as the area under the spectra between 435–485 nm. The FRET efficiency (E) was calculated as follows:

$$E=1-\frac{F_{DA}}{F_{D}}$$ (8)

The f ‘ value is defined as the ratio of FRET efficiency and the donor emission in the presence of the acceptor [38]:

$$f^{\prime }=\frac{E}{F_{DA}}$$ (9)

The relative f ‘ is derived by normalizing the f ‘ values to the value obtained at the lowest temperature [30, 37, 38].

### Coupled assay

The Mg^2+^-ATPase activity of HMM was measured with a Jasco V-550 spectrophotometer. The assay is based on a reaction in which the regeneration of hydrolysed ATP is coupled to the oxidation of NADH. The reaction buffer contains 100 mM KCl, 20 mM MOPS, 0.5 mM MgCl_2_, 0.5 mM ATP, 1 mM PEP, 0.5 mM EGTA, 5–10 μl PK (2 U/μl), 10 μl LDH (4 U / μl) in 60% glycerol, 0.15 mM NADH, pH 7.0. Following each cycle of ATP hydrolysis of 0.5 μM HMM (heavy-meromyosin) with or without 1 μM F-actin in the presence or absence of 1 μM Lmod2^FL^, the regeneration system consisting of phosphoenolpyruvate (PEP) and pyruvate kinase (PK) converts one molecule of PEP to pyruvate when the ADP is converted back to the ATP. The pyruvate is subsequently converted to lactate by L-lactate dehydrogenase (LDH) resulting in the oxidation of one NADH molecule. The assay measures the rate of NADH absorbance decrease at 340 nm, which is proportional to the rate of steady-state ATP hydrolysis. The constant regeneration of ATP allows monitoring the ATP hydrolysis rate over the entire course of the assay. The rate of ATP hydrolysis is calculated from the following equation:

$$\text{k}=-\frac{{\text{dA}}_{340}}{\text{dt}}{\text{x}\;\text{E}}_{\text{path}}^{-1}{\text{x}\;\text{M}}_{\text{ATP}}^{-1}$$ (10)

where k is the ATPase activity, A_340_ is the measured absorbance at 340 nm, t is the elapsed time, E_path_ is the molar absorption coefficient of NADH, M_ATP_ is the molarity of ATP, k values were corrected for the HMM concentrations.

## Results and discussion

### Intrinsic structural dynamics of cardiac Lmod2

**Fig 2:**
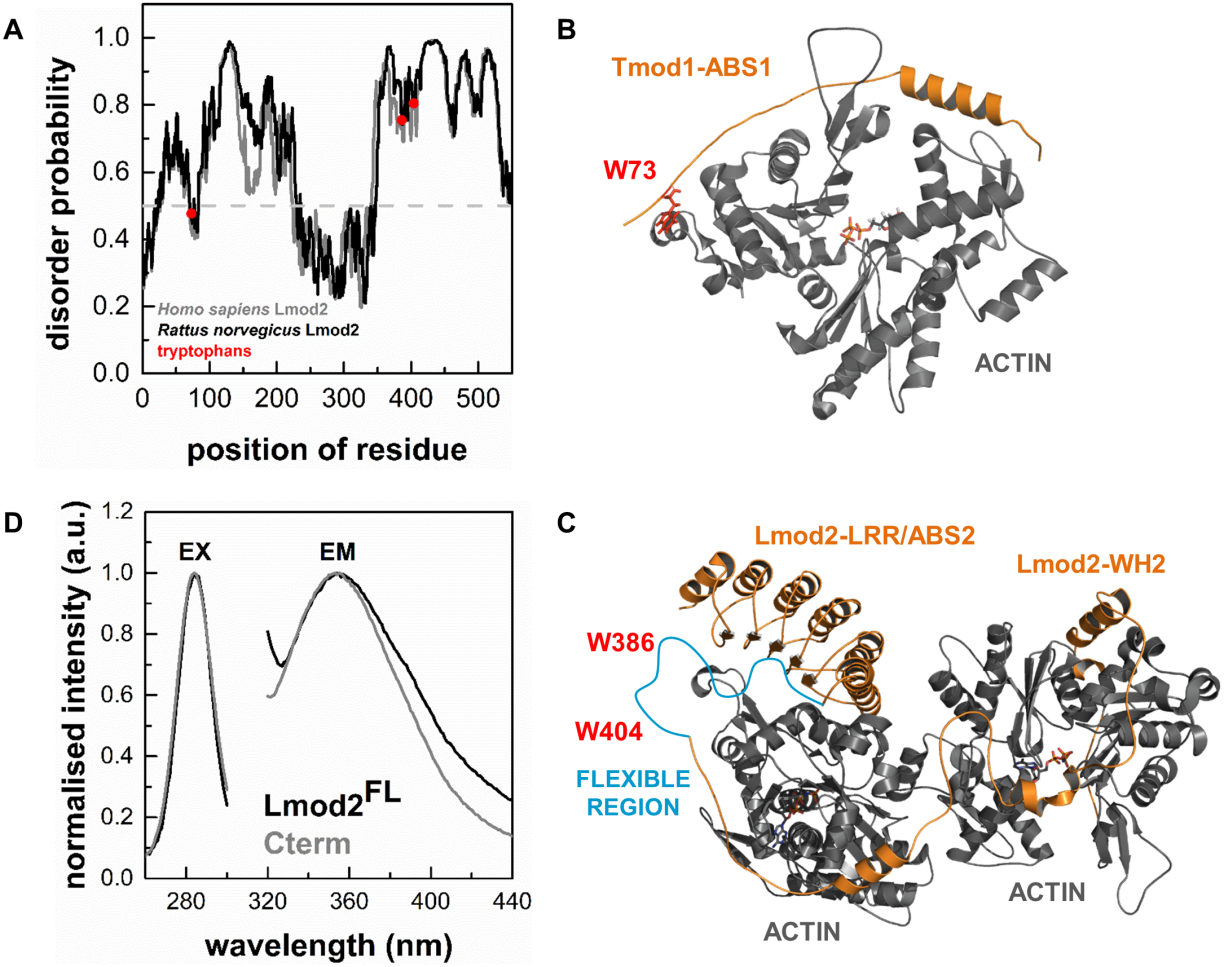
Intrinsic structural properties of Lmod2. (A) Bioinformatics analysis of Lmod protein sequences predicts intrinsically unstructured protein regions. The disorder probability in *Homo sapiens* Lmod2 and *Rattus norvegicus* Lmod2 (Uniprot accession numbers are Q6P5Q4 and A1A5Q0, respectively). The analysis was performed by IUPred [42, 43]. The tryptophan residues of *Rattus norvegicus* Lmod2 are highlighted by red. (B) Structural features of the interactions of Tmod1 with actin. The complex between Tmod1 ABS1 and actin is shown (PDB ID: 4PKG). The position of W73 of *Rattus norvegicus* cardiac leiomodin2 is predicted by multiple sequence alignment and highlighted by red. (C) Structural features of the interactions of Lmod2 with actin. The complexes between Lmod2 ABS2/LRR or WH2 actin binding sites and actin monomers are shown (PDB ID: 4PKG). The position of W386 and W347 (red) of *Rattus norvegicus* cardiac leiomodin2 are predicted to localize in a flexible protein region (blue) by multiple sequence alignment (PDB ID: 4RWT). This flexible region between residues 339–388 is missing from the structure of 4RWT. (D) Tryptophan fluorescence spectra of Lmod2. Excitation (EX) and emission (EM) spectra of the intrinsic tryptophans of Lmod2^FL^ (1 μM, black line) and Cterm (4 μM, grey line) were obtained at emission wavelength of 354 nm and excitation wavelength of 282 nm.

The crystal structure of the C-terminal regions of human Lmod2 in complex with actin was recently reported [13], which shows that Lmod2 possesses well-structured regions, as well as highly flexible elements, which are missing from the structure. In agreement with this, our bioinformatics analysis predicts structural elements in Lmod2, which are characterised by relatively large disorder probability, characteristic for intrinsically disordered protein regions (IDRs) (disorder probability > 0.5, Fig 2A) [39–41]. *Rattus norvegicus* leiomodin2 contains three tryptophans (W73, W386 and W404), which are located in segments with high predicted disorder (Fig 2A). Multiple sequence alignment predicts that the W73 of cardiac Lmod2 is located at same position as L71 in human tropomodulin1. W386 and W404 are conserved between *Rattus norvegicus* Lmod2 and human Lmod1 (corresponding residues are W347 and W365 in the human protein, respectively). L71 lays on a flexible chain of actin binding site ABS1 in Tmod1 (PDB ID: 4PKG [21]). The structural similarities implicate that the W73 residue in Lmod2 would be localised on a flexible chain of the actin binding domain ABS1 (Fig 2B) [26]. The C-terminal tryptophan of human Lmod1 are found in a highly flexible region (residues 339–388) that is missing from its crystal structure (PDB ID: 4RWT [13], Fig 2C), suggesting a similar structural environment for W386 and W404 in Lmod2.

We used these intrinsic fluorophores to characterise the structural dynamics of full length cardiac leiomodin2 (Lmod2^FL^) and its truncated C-terminal segment (Cterm) in fluorescence spectroscopic approaches. First, to describe the spectral properties of tryptophan residues we recorded their emission and excitation spectra (Fig 2D). The spectra obtained for Lmod2^FL^ and Cterm showed identical excitation maxima at ~ 282 nm, and the wavelength corresponding to the emission maxima was also close for the two proteins (~ 354 nm). This indicates that the microenvironments of the tryptophan residues were similar in the full length leiomodin2 and its C-terminal fragment. We carried out the subsequent spectroscopic measurements using the above identified maximum excitation and emission wavelengths.

To describe the flexibility of the protein matrix we also measured the steady-state anisotropy (r). The values were 0.083 ± 0.015 and 0.038 ± 0.005 for Lmod2^FL^ and Cterm, respectively. Taking into account the size of the proteins (Lmod2^FL^: 62 kDa and Cterm: 19.5 kDa) and the theoretical maximum of the steady-state anisotropy for a perfectly immobilised system (0.4), these are relatively low anisotropy values, indicating that the tryptophans are located in unstructured and flexible protein regions in both Lmod2^FL^ and Cterm. To further explore the structural properties of leiomodin we also carried out time-dependent anisotropy decay experiments. The anisotropy decay was fitted with double exponential functions and the fits provided two rotational correlation times for each protein (data not shown). The shorter correlation times were sub-nanosecond values. Although, due to the resolution limits of the experiments the proper interpretation of the actual values is difficult, we attributed these short correlation times to the wobbling motion of the tryptophan in their flexible protein environment [31, 44–46]. The longer rotational correlation time was 35.9 ± 6.5 ns and 6.9 ± 1.6 ns for Lmod2^FL^ and Cterm, respectively. These values characterise the rotational diffusion of the entire protein in both cases. Lmod2 (62 kDa) is a larger protein than Cterm (19.5 kDa), which explains the longer correlation times obtained for Lmod2^FL^. In these experiments we observed that the probes located in a flexible protein region, where little limitation was posed on its wobbling rotational motion.

In conclusion, our experimental data suggests that the tryptophan residues of Lmod2 are located in disordered and flexible regions, consistently with the structural and bioinformatics prediction.

### The effects of purified cardiac leiomodin2 on actin assembly dynamics

**Fig 3:**
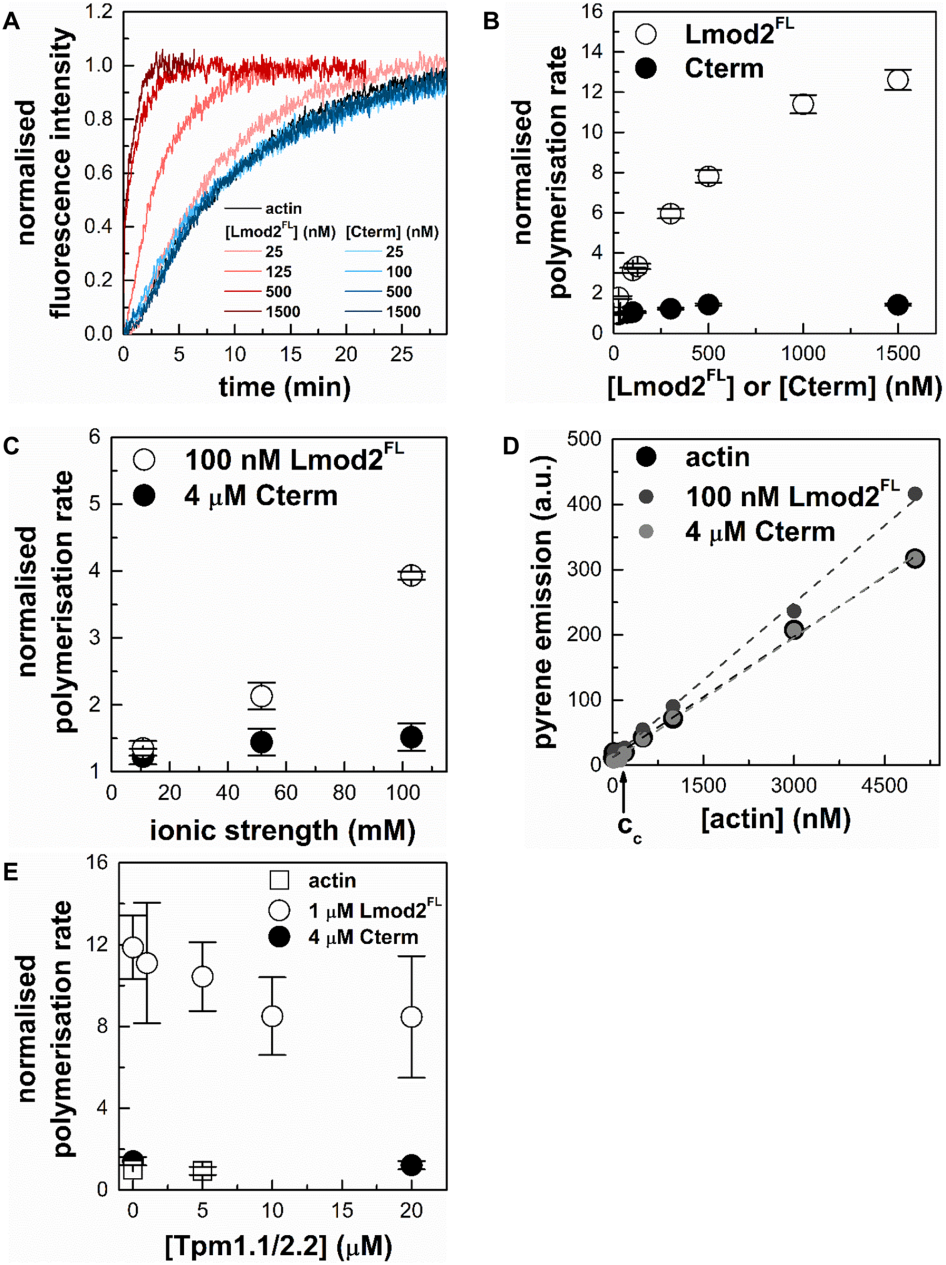
The effects of purified cardiac leiomodin2 on actin assembly dynamics. (A) Effects of cardiac Lmod2 and Cterm on the polymerisation kinetics of actin. The kinetics of actin assembly (4 μM, containing 5% pyrene labelled actin) in the absence and presence of different concentrations of Lmod2^FL^ or Cterm. Pyrene fluorescence was measured at excitation and emission wavelengths of 350 nm and 404 nm, respectively. Salt conditions: 100 mM KCl, 2 mM MgCl_2_. (B) Cardiac Lmod2 influences actin polymerisation in a concentration dependent manner. Polymerisation rates were determined from the slope of the pyrene curves (shown on panel (A)) at 50% polymerisation and normalised by the rate measured for spontaneous actin assembly. The actin polymerisation rates are plotted as a function of Lmod2^FL^ (empty circles) and Cterm (filled circles) concentrations. (C) Ionic strength dependence of the effects of cardiac Lmod2 and Cterm on actin polymerisation. Pyrene-actin (4 μM, 5% labelled) was polymerised in the absence or presence of 100 nM Lmod2^FL^ (empty circles) or 4 μM Cterm (filled circles) under low (10 mM KCl, 0.5 mM MgCl_2_), medium (50 mM KCl, 1 mM MgCl_2_) and high (100 mM KCl, 2 mM MgCl_2_) salt conditions. Normalised polymerisation rates were derived as described above and plotted as a function of ionic strength (Eq 4). (D) The effect of cardiac Lmod2 on the critical concentration of actin assembly. Pyrene intensities as a function of actin concentration were measured in the absence (black circles) or presence of 100 nM Lmod2^FL^ (grey circles) or 4 μM Cterm (light grey circles) under high salt conditions (100 mM KCl, 2 mM MgCl_2_). Actin (5% pyrene labelled) concentrations were 30; 50; 100; 300; 500; 700 nM and 1; 3; 5 μM. The critical concentrations were determined by using Eq 5 and were found to be 120 ± 83 nM in the absence of Lmod2^FL^ and 117 ± 68 nM and 132 ± 58 nM in the presence of Lmod2^FL^ and Cterm, respectively. Dashed lines in the corresponding colour show the fit to the data. (E) Skeletal tropomyosin (Tpm1.1/2.2) reduces the polymerisation activity of Lmod2. Pyrene-actin (4 μM, 5% labelled) was polymerised in the absence (empty squares) or presence (empty circles) of 1 μM Lmod2^FL^ or 4 μM Cterm (filled circles) under high salt conditions (100 mM KCl, 2 mM MgCl_2_) in the presence of different concentrations of skeletal muscle tropomyosin. Data are presented as mean ± SD (n = 3).

To test whether our proteins are functional, pyrene actin polymerisation assays were carried out in the absence or presence of Lmod2^FL^ or Cterm (25–1500 nM) (Fig 3A). In these experiments the increase of the pyrene fluorescence reported on the increase of the actin filament concentration. The rate of actin polymerisation increased in the presence of Lmod2^FL^ (Fig 3A and 3B), in agreement with previous reports [7, 8, 13]. This increase was Lmod2^FL^ concentration dependent and followed a saturation curve, its maximal value being ~ 12 times larger in the presence than in the absence of Lmod2^FL^. This substantial increase of the polymerisation rate indicated that Lmod2^FL^ can effectively accelerate actin assembly. Cterm did not influence significantly actin polymerisation in the concentration range tested (Fig 3A and 3B).

Protein-protein interactions often depend on the ionic strength. To describe whether the effect of leiomodin on actin polymerisation is salt dependent we measured the rates of actin polymerisation under low salt (0.5 mM MgCl_2_, 10 mM KCl), medium salt (1 mM MgCl_2_, 50 mM KCl) or high salt (2 mM MgCl_2_, 100 mM KCl) conditions in the absence and presence of Lmod2^FL^ (100 nM) or Cterm (4 μM). We found ionic strength dependence in all cases (Fig 3C), although the salt dependence was much pronounced for Lmod2^FL^ than for Cterm. In the presence of Lmod2^FL^ the increase in the rate of actin polymerisation was 1.2 fold at low salt and 4 fold at high salt conditions.

To further investigate the effects of Lmod2 on actin dynamics, we measured the critical concentration of actin in the absence and presence of Lmod2^FL^ and Cterm (Fig 3D). Pyrene labelled actin was incubated under polymerising conditions at various concentrations and then the pyrene fluorescence intensity was measured. The plot of these intensities as the function of the actin concentration showed a breaking point, corresponding to the critical concentration (Fig 3D). In the absence of Lmod2^FL^ the critical concentration was 120 ± 83 nM at high salt conditions, in good agreement with the well-established value [30, 47]. In the presence of 100 nM Lmod2^FL^ or 4 μM Cterm the critical concentration was not significantly affected (117 ± 68 nM and 132 ± 58 nM, respectively). The close agreement between these critical concentration values indicated that the amount of actin that was polymerised in the absence and presence of Lmod2 proteins was similar. The lack of the shift in the breaking point is in agreement with the observations that Lmod2 does not affect barbed end dynamics, consistently with its pointed end localisation [7, 8, 13].

The above observations indicate that the Cterm, possessing a single actin-binding WH2 domain is not sufficient for enhancing actin polymerisation. The efficient actin assembly promoting activity of Lmod2 requires the N-terminal actin binding domains. Our data are in agreement with previous reports and show that *Rattus norvegicus* cardiac Lmod2 behaves similarly to the human protein in terms of actin dynamics regulation [7, 14].

### Tropomyosin influences the Lmod2-mediated actin polymerisation

In muscle cells actin dynamics is regulated by a large repertoire of ABPs [1, 2]. An essential member of this family in muscle cells is tropomyosin that decorates the side of actin filaments [48, 49]. To extend our investigations, we tested whether the presence of skeletal muscle tropomyosin (Tpm1.1/2.2) can change the effect of leiomodin on actin polymerisation. The polymerisation kinetics of pyrene labelled actin was monitored in the presence of Lmod2^FL^ (1 μM) or Cterm (4 μM) and in the presence of various concentrations (0–20 μM) of tropomyosin (Fig 3E). Tropomyosin did not affect actin polymerisation in the absence of Lmod2^FL^. In the presence of Lmod2^FL^ tropomyosin induced a concentration dependent decrease in the rate of actin assembly. At the highest concentration of tropomyosin (20 μM) the rate of Lmod2^FL^ mediated actin polymerisation was decreased to ~ 70% of its value measured in the absence of tropomyosin. Statistical analysis revealed that this change was not significant (p = 0.2017). We found that tropomyosin did not influence significantly actin assembly in the presence of Cterm (Fig 3E).

In conclusion, skeletal muscle tropomyosin influences Lmod2 mediated actin polymerisation. Our observations suggest, that the inhibitory effect of tropomyosin on leiomodin mediated actin polymerisation could appear due to enhanced capping activity of the N-terminal of Lmod2, consistently with recent findings [8].

### Lmod2 affects the structure of filamentous actin

**Fig 4:**
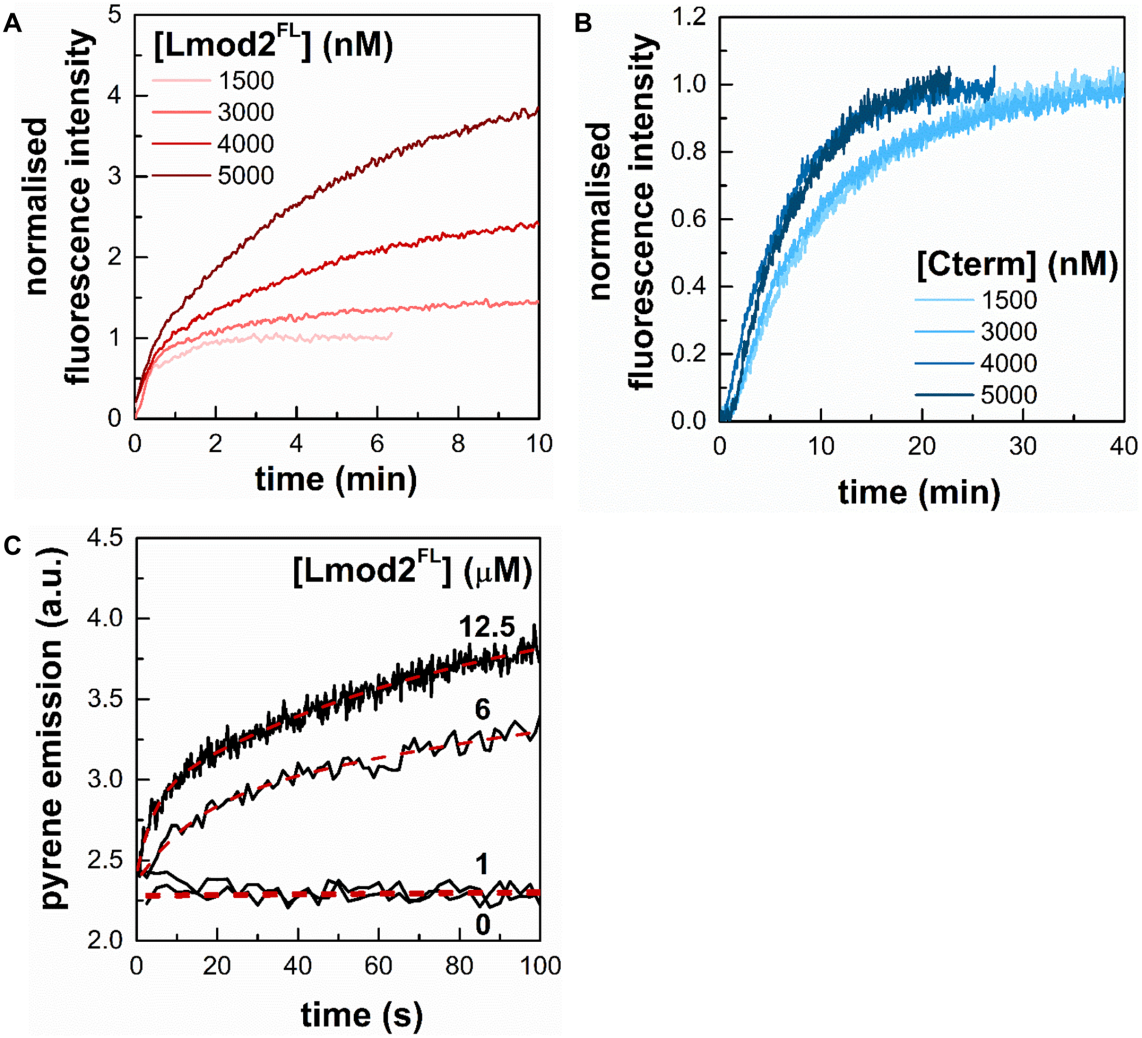
Lmod2 influences pyrene emission in actin filaments. (A) Polymerisation kinetics of actin assembly (4 μM; 5% labelled) in the presence of Lmod2^FL^ show increased saturation of pyrene fluorescence emission in a concentration dependent manner. (B) Cterm does not affect the steady-state value of pyrene actin fluorescence. The actin concentration was 4 μM, containing 5% labelled actin. (C) Rapid kinetics measurements of the time dependent change in pyrene F-actin fluorescence. Stopped-flow measurements of the kinetics of pyrene fluorescence emission of prepolymerised F-actin (1 μM, 5% pyrene labelled) in the absence or presence of different concentrations of Lmod2^FL^ (1 μM, 6 μM and 12.5 μM, as indicated). Salt conditions: 100 mM KCl, 2 mM MgCl_2_. Dashed lines show the fit using Eq 7.

In the above experiments the pyrene intensity after saturation was increased in a Lmod2^FL^ concentration dependent manner, addition of 5 μM Lmod2^FL^ resulted in a ~ 4 fold increase (Fig 4A). The PDB structures of 4PKG and 4RWT clearly show that ABS1 and helical domains from Cterm both bind nearby of Cys374 on actin which can affect the freedom and fluorescence emission of fluorophores on cysteine. Considering that the actin polymerisation assays were carried out with 4 μM actin and assuming that the value of the critical concentration is 100–150 nM, (Fig 3D and [23, 30, 47]), we estimate that approximately 95% of actin polymerises in the absence of leiomodin. Therefore, the large (~ 4 fold) increase in the maximum fluorescence intensity observed with Lmod2^FL^ cannot be explained by assuming that more actin was in polymer form in the presence of Lmod2^FL^ than in its absence. Considering the above observations, we interpret our data by proposing that Lmod2^FL^ interacts with actin filaments and its binding substantially increases the fluorescence quantum yield and thus the intensity of pyrene.

To further confirm the fluorescence increase of leiomodin binding we carried out rapid kinetic stopped-flow experiments using prepolymerised actin filaments, where the optical properties of the solution are the same before and after the binding of leiomodin, therefore eliminating the possible artefacts arising from the mixing of buffers. We wanted to characterise a second order binding process therefore the leiomodin concentration was designed to cover a broad concentration range, where the faster binding rates could also be determined. These rapid kinetic assays showed that Lmod2^FL^ increased the fluorescence emission of 1 μM pyrene labelled actin filaments in a concentration dependent manner (Fig 4C). In subsequent experiments we found that Lmod2^FL^ can also amplify the emission of IAEDANS labelled actin filaments as well (data are not shown). These observations suggest that Lmod2 affects the structure of actin filaments, and could serve as a basis for further spectroscopic and kinetic experiments.

### Lmod2 binds to the side of actin filaments

The fluorescence quantum yield of actin bound probes increased in the presence of leiomodin. One explanation for this observation is to consider that leiomodin2 through its pointed-end binding ability [7, 8, 14] induces structural changes in F-actin, which affects the fluorescence emission of actin-bound probes, as reported for other ABPs; e.g. gelsolin and formins [30, 31, 50, 51]. Lmod2, besides end binding, can bind to the sides of actin filaments, i.e. it can decorate the filaments along their length [28]. This way Lmod2 could directly alter the quenching processes that determine the fluorescence intensity of the probes.

**Fig 5:**
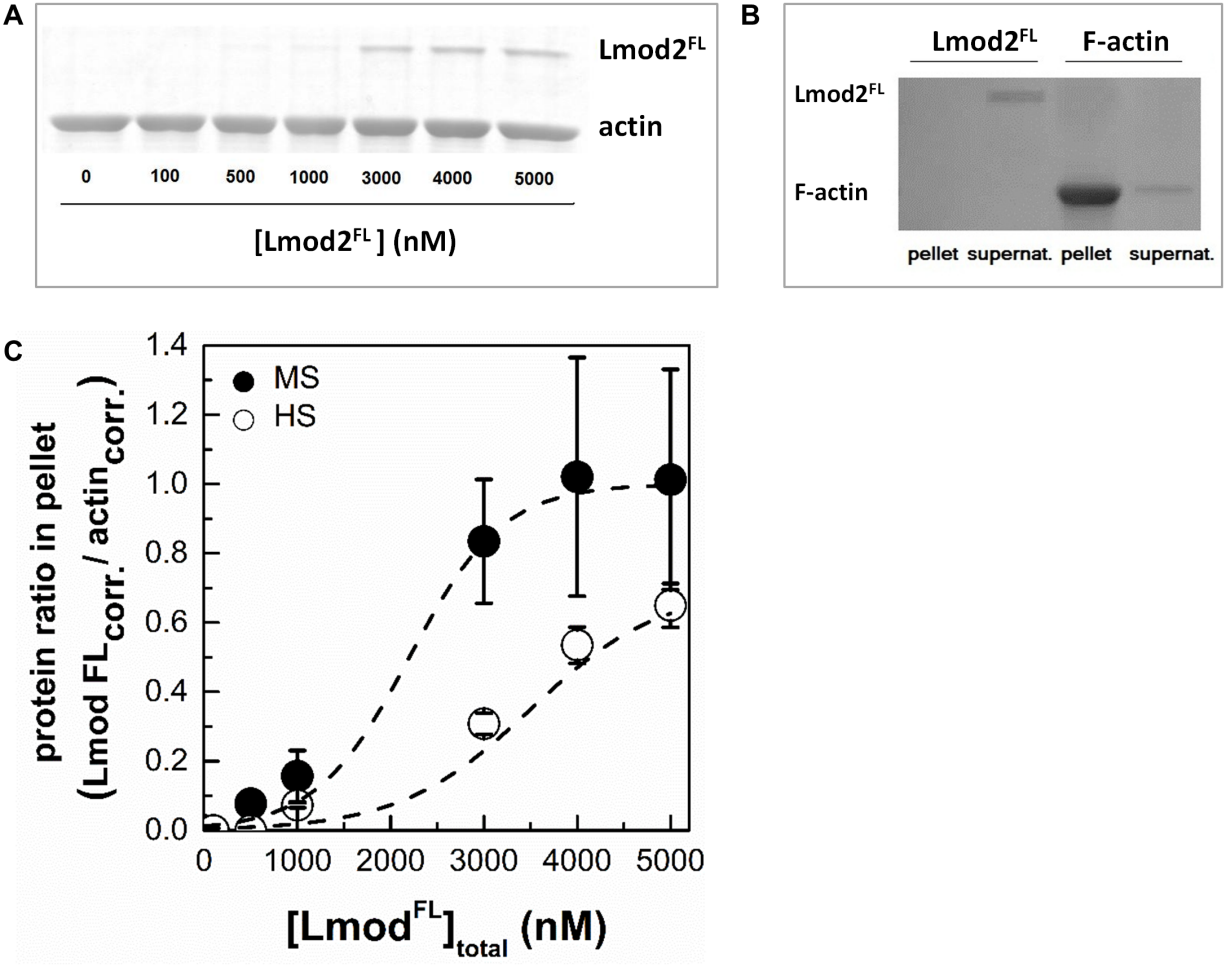
Cardiac leiomodin2 binds to the sides of actin filaments. (A) In high-speed cosedimentation assays F-actin (4 μM) and Lmod2^FL^ (0–5 μM) was incubated under high salt conditions (100 mM KCl, 2 mM MgCl_2_) and centrifuged at 258.000 g for 30 min at room temperature. The pellets were analysed by SDS-PAGE. Representative Coomassie stained SDS-PAGE gel of the pellets is shown. (B) Lmod2^FL^ (5 μM) does not sediment in the absence of actin. Leiomodin was centrifuged in the absence of F-actin (left two columns) or F-actin (5 μM) was pelleted in the presence of Lmod2^FL^ (right two columns). Pellets and supernatants are indicated on the figure. (C) Actin-Lmod2^FL^ ratios in pellet were calculated from SDS-PAGE analysis of under high salt (HS, 100 mM KCl, 2 mM MgCl_2_) and medium salt (MS, 50 mM KCl, 1 mM MgCl_2_) conditions, and plotted as the function of Lmod2^FL^ concentration. Dashed lines show the fit using Eq 6. Data are presented as mean ± SD (n = 3).

To test how does Lmod2^FL^ bind to the sides of actin filaments we carried out high-speed co-sedimentation experiments (Fig 5). We tested only Lmod2^FL^ for this ability, since Cterm failed to increase the pyrene signal in spectroscopic experiments (Fig 4). In these experiments actin (4 μM) and Lmod2^FL^ (0–5 μM) were coincubated and centrifuged. The pellets and supernatants were analysed by SDS-PAGE. We observed that increasing the total concentration of leiomodin resulted in increased leiomodin concentration in the pellets (Fig 5A). In control experiments, Lmod2^FL^ did not appear in the pellet in the absence of actin (Fig 5B). Since the concentration of pointed ends of micromolar actin filaments is a few nanomolar, the amount of leiomodin detected in the pellets was much greater than that expected from only filament end binding. Therefore, our observation can only be explained by assuming that Lmod2^FL^ bound to the side of actin filaments and pelleted with them as a complex. The binding of Lmod2 to F-actin was salt sensitive, its strength was decreased by increasing ionic strength (from medium salt to high salt conditions) (Fig 5C). In conclusion, in addition to its pointed end binding ability, leiomodin binds to the sides of actin filaments *in vitro*.

### Structural consequences of F-actin binding by Lmod2

**Fig 6:**
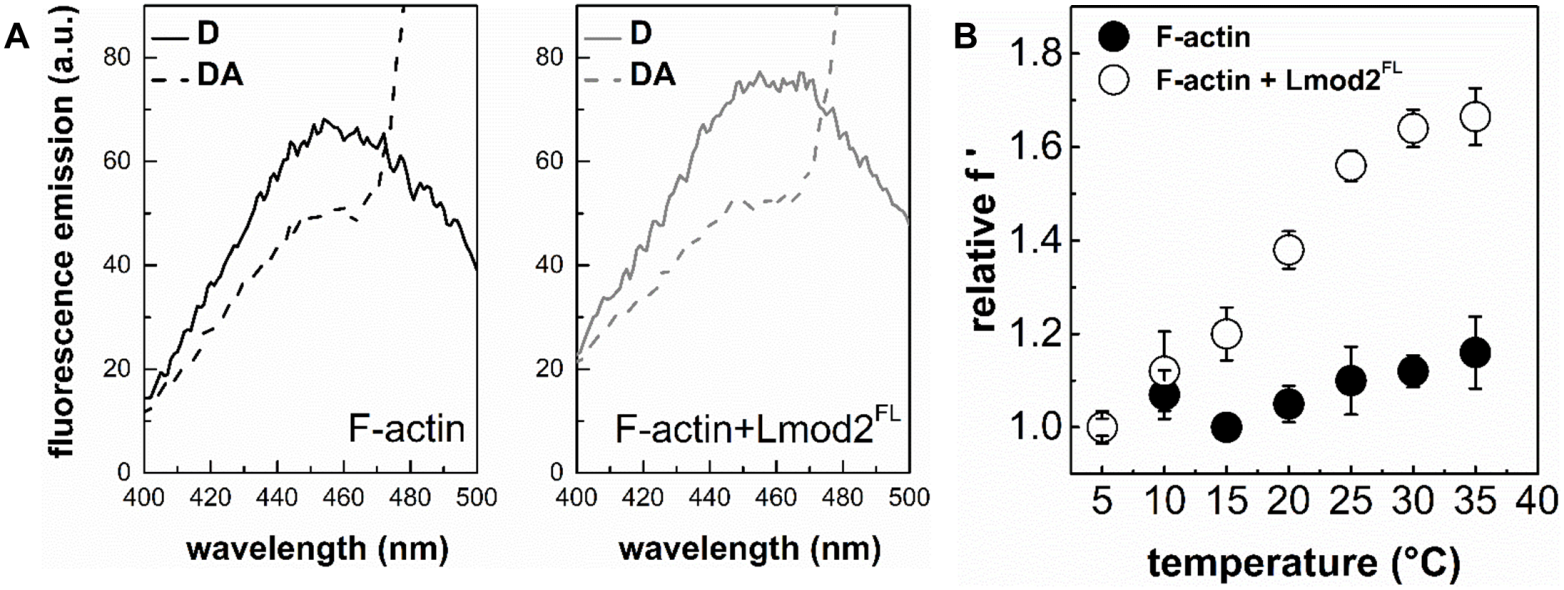
The effect of leiomodin2 on the flexibility of actin filaments. (A) Emission spectra of IAEDANS (D, donor) in the absence and presence of IAF (A, acceptor), and in the absence and presence of Lmod2^FL^. The actin filament concentration was 4 μM. Note the acceptor induced decrease in the donor emission due to FRET. (B) Temperature-dependent FRET measurements were performed on IAEDENS-IAF actin filaments (4 μM) in the absence and presence of different concentrations of Lmod2^FL^. FRET efficiencies calculated from the emission spectra (representative spectra are shown on panel (A)) were normalised by FRET efficiency measured at the lowest temperature (relative f ‘, Eq 9) and plotted as a function of temperature. Data obtained in the absence (filled circles) or presence (empty circles) of Lmod2^FL^ (5 μM) are shown. Data are presented as mean ± SD (n = 3).

The above observations suggest that by binding to actin filaments Lmod2 can affect the structural properties of F-actin. To address this issue, inter-monomer FRET measurements were carried out with double labelled actin filaments [30]. Actin monomers were labelled separately with IAEDANS as a donor or with IAF as an acceptor. The two pools of labelled actin monomers were mixed and then co-polymerised. Using this strategy, the energy transfer between neighbouring actin protomers in the filament, i.e. the inter-monomer FRET can be investigated. As expected, we observed a decrease in the donor emission in the presence of the acceptor (Fig 6A). The inter-monomer FRET efficiency within filaments (4 μM) in the absence of Lmod2 was found to be 26%. Addition of Lmod2^FL^ (5 μM) resulted in an increase of the inter-monomer FRET efficiency to 32%. We interpret this increase as a consequence of filament binding of leiomodin. The first possible explanation is that Lmod2^FL^ binding changes the radial coordinate of the fluorescently labelled residue, whereas the second one is that it changes the flexibility of actin filaments [30, 37, 38]. In the first one would assume that the fluorophores moved closer to the longitudinal axis of the filament, leading to a more compact and probably stiffer structure and thus to an increase in the FRET efficiency. In the second case, the binding of leiomodin would make the structure of the filament more dynamic, thereby increasing the frequency and/or the amplitude of the relative oscillations of the fluorophores, resulting in larger FRET efficiencies [30, 38, 46]. To test which of the two explanations are valid we carried out temperature-dependent FRET experiments, where the determined FRET efficiencies are normalized by the fluorescence intensity of the donor measured in the presence of acceptor. The resulted temperature dependence of the normalized energy transfer provides information on the flexibility of the protein matrix between the donor and the acceptor [38]. The theory has been shown to be valid even if multiple fluorophore systems are applied, e.g. when one donor is in FRET interaction with more than one acceptor [52], as it is the case here. Our normalized FRET data showed steeper temperature dependence in the presence than in the absence of leiomodin (Fig 6B), indicating that the structure of the filaments become more flexible upon the binding of Lmod2^FL^. Considering that the Lmod2^FL^ induced effects on the fluorescence emission of the probes, as well as on the inter-monomer FRET efficiency were observed at relatively large protein concentrations (~ 5 μM), we propose that these effects originate mainly from side-binding and that the binding of Lmod2^FL^ affects the structural dynamics of actin filaments.

### Functional consequences of F-actin binding by Lmod2

**Fig 7:**
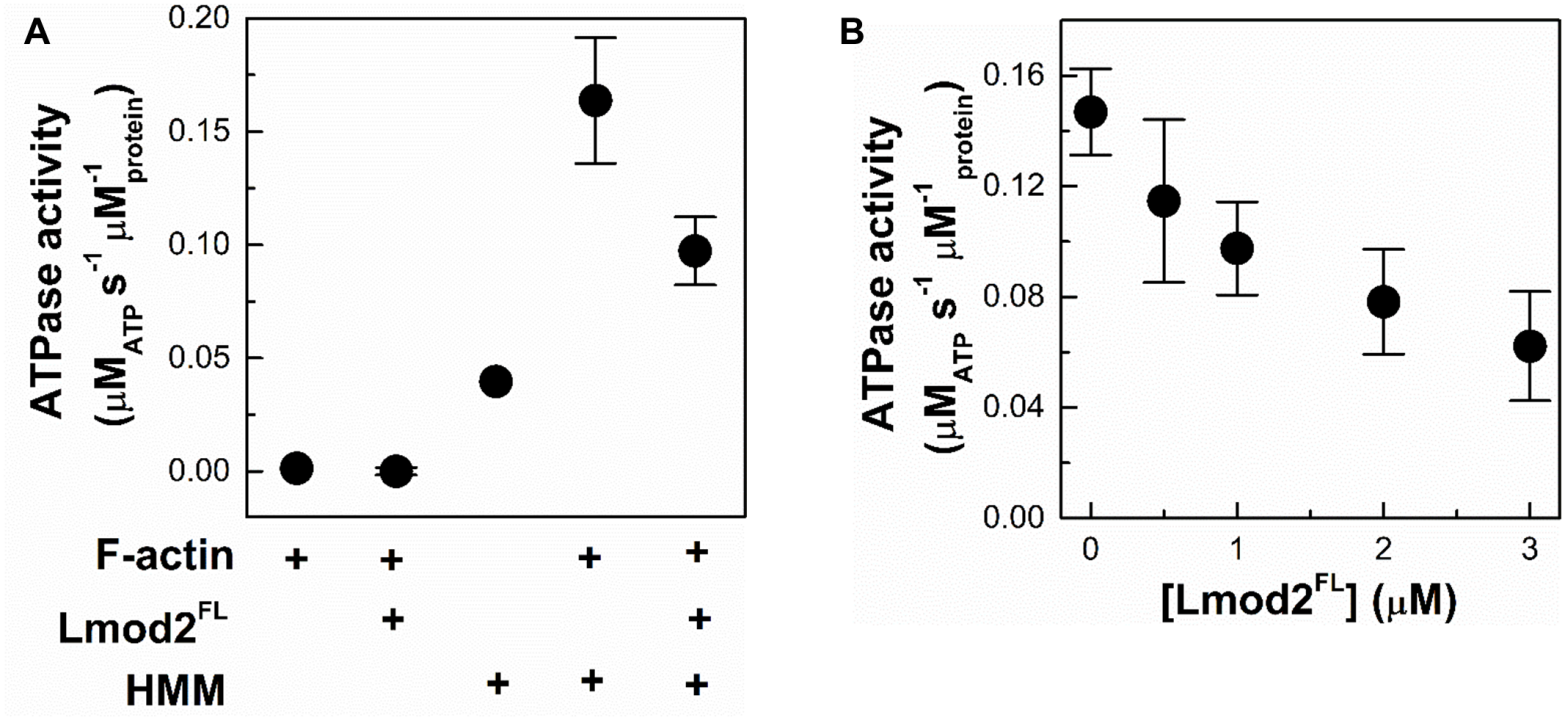
Functional consequences of F-actin binding by Lmod2. (A) The effect of Lmod2^FL^ on the Mg^2+^-ATPase activity of HMM measured with coupled assay. The Mg^2+^-ATPase activity of HMM (0.5 μM) in the absence and presence of actin and/or Lmod2^FL^ was measured under low salt conditions (10 mM KCl, 0.5 mM MgCl_2_). (B) The Mg^2+^-ATPase activity of HMM (0.5 μM) in the presence of F-actin (1 μM) as the function of Lmod2^FL^ concentration. Data are presented as mean ± SD (n = 3).

Myosin is a prominent actin-binding protein playing central role in muscle functioning. To further elaborate on the functional consequences of the side-binding ability of Lmod2, we investigated whether the ATPase activity of skeletal muscle myosin II is altered by leiomodin. A truncated form of skeletal muscle myosin, the double-headed heavy meromyosin (HMM) was used in these studies. The Mg^2+^-ATPase activity of HMM was measured by using a coupled assay in the absence or presence of actin filaments (1 μM) and / or leiomodin (1 μM). In control experiments, in the absence of HMM, ATPase activity was not detected for either F-actin or Lmod2^FL^ (Fig 7A). The basal and the F-actin (1 μM) activated Mg^2+^-ATPase activities of HMM (0.5 μM) were 0.04 μM_ATP_ s^-1^ μM_protein_^-1^ and 0.164 μM_ATP_ s^-1^ μM_protein_^-1^, respectively, consistent with previous data [53]. We observed that the actin-activated Mg^2+^-ATPase activity of HMM decreased in the presence of Lmod2^FL^ in a concentration-depended manner (Fig 7) reaching ~ 40% of its original value at 3 μM Lmod2^FL^ concentration (Fig 7B).

The above observations show that by binding to actin filaments Lmod2^FL^ can regulate the ATPase activity of myosin. This can be explained either by direct or indirect structural effects. Lmod2 may directly block the ATP binding cleft on HMM or the Lmod2 binding site may overlap with that of myosin II. Alternatively, a mechanism based on allosteric structural changes in actin filaments can be considered; the binding of Lmod2 to filaments may induce long-range structural changes, which may affect myosin II and F-actin interaction. Several ABPs, including formins, gelsolins and ADF-cofilins and myosin were reported to impose allosteric effects to actin filaments upon binding [30, 31, 50, 51, 54–56]. Allosteric regulation upon cooperative structural changes in actin filaments were shown to contribute to the mutually exclusive binding of myosin and ADF/cofilin proteins [57].

## Conclusions

In this work we showed that *Rattus norvegicus* cardiac leiomodin2 possesses similar structural and functional features as human leiomodin2. *Rattus norvegicus* Lmod2 contains highly flexible, intrinsically disordered regions, similarly to its Tmod homologues and other Lmods [13, 21]. In terms of actin dynamics, we showed that *Rattus norvegicus* Lmod2 influences the rate of actin polymerisation in an ionic strength dependent manner (Fig 3). This indicates that leiomodin2 may play a role in the regulation of actin filament generation in locations where tropomyosin and matured actin filaments are present, and new actin filaments are not needed.

Apart from its already known colocalisation with filament ends, Lmod2 was detected along the length of thin filaments in rat cardiomyocytes, as well as in M-lines [14, 28]. Consistently with these observations, we demonstrated that Lmod2 can bind to the sides of actin filaments *in vitro* (Fig 5). The binding of leiomodin to actin filaments made the structure of the filaments more flexible (Fig 6). As a functional consequence, Lmod2 decreased the actin enhanced Mg^2+^-ATPase activity of myosin upon F-actin binding (Fig 7). These novel interactions of leiomodin have functional implications. The localisation of leiomodin in living muscle cells is not limited to the ends of the thin filaments, the presence of Lmod along the actin filaments, as well as in regions where myosin II is localized indicates that Lmod may influence acto-myosin activity in the cellular context. Leiomodin by reducing the activity of myosin, as we detect in our experiments even in the presence of lower amount (≤ 1 μM) of Lmod2, may decrease the generated force during the interaction of thin and thick filaments. Interestingly, the inhibition of myosin II activity with blebbistatin resulted in the delocalisation of leiomodin in cultured cardiomyocytes, which was interpreted as a lack of need for *de novo* actin polymerization considering the *in vitro* nucleation activity of Lmod2 [28]. Blebbistatin preferentially binds to the weak-binding ADP.P_i_ intermediate of myosin II and slows down phosphate release, thereby it blocks myosin heads in their low-affinity complexes [58]. This way blebbistatin alters the equilibrium between the actin bound and unbound myosin populations. Considering this, we propose an alternative explanation for the blebbistatin-induced Lmod2 dissociation from actin filaments. Active and contractile sarcomeres require the actin filament localization of Lmod2 to properly tune the force-generation by the acto-myosin system. The inactivity of myosin II upon blebbistatin treatment results in uncontractile machinery, which does not require the optimization of acto-myosin interaction. This may result in the dissociation of Lmod2 from actin filaments. Our findings on the Lmod2 dependent activity of myosin II can supplement the recently published new model of cyclic activities of Lmod in sarcomeric functions and provides an *in vivo* support for the interpretation of our results [59].

Considering that previous data indicated important roles of leiomodin in the developing heart [9], the regulation of the acto-myosin cross-bridge activity by leiomodin maybe essential in developing heart muscle cells, where larger than optimal forces may perturb the proper formation of the sarcomeric structure.
